# Enhancing medical students' communication skills: development and evaluation of an undergraduate training program

**DOI:** 10.1186/1472-6920-12-16

**Published:** 2012-03-24

**Authors:** Maria C Hausberg, Anika Hergert, Corinna Kröger, Monika Bullinger, Matthias Rose, Sylke Andreas

**Affiliations:** 1 Department of Medical Psychology, University Medical Centre Hamburg-Eppendorf, Martinistraße 52, 20246 Hamburg, Germany; 2 Department of Psychosomatic Medicine and Psychotherapy, University Medical Centre Hamburg-Eppendorf, Martinistraße 52, 20246 Hamburg, Germany

## Abstract

**Background:**

There is a relative lack of current research on the effects of specific communication training offered at the beginning of the medical degree program. The newly developed communication training "Basics and Practice in Communication Skills" was pilot tested in 2008 and expanded in the following year at the University Medical Centre Hamburg-Eppendorf in Germany. The goal was to promote and improve the communicative skills of participants and show the usefulness of an early offered intervention on patient-physician communication within the medical curriculum.

**Methods:**

The students participating in the project and a comparison group of students from the standard degree program were surveyed at the beginning and end of the courses. The survey consisted of a self-assessment of their skills as well as a standardised expert rating and an evaluation of the modules by means of a questionnaire.

**Results:**

Students who attended the communication skills course exhibited a considerable increase of communication skills in this newly developed training. It was also observed that students in the intervention group had a greater degree of self-assessed competence following training than the medical students in the comparison group. This finding is also reflected in the results from a standardised objective measure.

**Conclusions:**

The empirical results of the study showed that the training enabled students to acquire specialised competence in communication through the course of a newly developed training program. These findings will be used to establish new communication training at the University Medical Centre Hamburg-Eppendorf.

## Background

Empirical findings show that both physicians and patients benefit from the effective use of communication skills. Studies have demonstrated that physicians' communication skills lead to greater therapy adherence [1] and overall satisfaction with care [2] among patients. Another effect frequently described in the literature is a decrease in patients' distress and susceptibility to symptoms of depression or anxiety [3-5]. Moreover, a decrease in health care utilisation related to physicians' good communication practises has recently been demonstrated [6].

These findings underline the importance of good communication techniques in medical consultations and indicate the need to provide future medical doctors with training in these skills. The first descriptions of communication training emerged in the early 1970s [7], and the subject is now well established in most medical schools across the US [8,9] and the UK [10,11], as well as some other European countries [12,13]. However, few studies have been published on communication training for medical students in German-speaking countries, indicating that these programs are yet to be established [14-17]. This assumption is also reflected in the results from a survey of medical school graduates at seven German universities. In this study, the vast majority of participants rated communication skills essential for their future professional careers, but they also noted that this area revealed the most striking deficits [18]. The significance of communication and social competencies in medical education across German-speaking countries has been emphasised in a recently published consensus statement [19]. The authors provide a comprehensive set of competencies and educational objectives for teaching communication in undergraduate medical education to support the nationwide implementation of these issues in all medical schools.

There is evidence that students' communication skills deteriorate during their clinical years [20] and teaching these skills is neglected during those years [21]. Therefore, one of the major aims of reformed medical curricua is to design and provide training for medical students that integrates knowledge and competencies through the early implementation of clinical experiences. Compared to the widespread research on postgraduate communication training, empirical results examining training within early medical studies are less common. Most publications involve descriptive work that introduces new concepts [9,22] or explores students' needs and perceptions [23,24]. Nevertheless, there is some evidence that communication skills training offered to students early in the curriculum is worthwhile [25-27].

The purpose of this study was to assess the effectiveness of newly developed communication training for first-year medical students to improve their knowledge and skills and to identify vital areas for implementation in the reform curriculum.

## Methods

The exact meaning of "communication skills" remains unclear, and detailed information on the content of communication skills education is often lacking [28]. Cegala and Lenzmeier Broz [28] reviewed 26 studies published since 1990 and found little consistency in definitions of communication skills in these studies. These authors note that, in many cases, the instruments used to assess communication effects miss the target of the intervention. We attempt to address this issue in the present study by carefully examining the literature on the assessment of communication skills to identify an appropriate measure for our training issues. To address the inconsistency in definitions, we use the Kalamazoo Consensus Statement [29] as a framework for our intervention. Published in 2001 by a group of representatives from major medical education and professional organisations, this statement provides a list of essential elements in patient-physician communication: (1) build the doctor-patient relationship; (2) open the discussion; (3) gather information; (4) understand the patient's perspective; (5) share information; (6) reach agreement on problems and plans; and (7) provide closure. We rely on the Kalamazoo statement because an equivalent for German-speaking countries had not been published at the time our intervention was planned.

### Design and participants

At the University Medical Centre Hamburg-Eppendorf, an interdisciplinary team of faculty members is working on an overall reform of the curriculum based on the Bologna process, the implementation of a university education system consisting of bachelor's and master's degrees that are comparable throughout Europe. This reform will also include the revision of previous methods of teaching communication. In the course of this process, we developed and evaluated existing communication training to gain knowledge about its effective components as well as students' needs and preferences.

The communication-training module "Basics and Practise in Communication Skills" was embedded in a newly developed supplementary qualification for students in psychosocial-medicine at the University Medical Centre Hamburg-Eppendorf in Germany. We offered the newly developed training to a group of students who agreed to take part in addition to their regular studies (psychosocial-medicine students = PMS). The concept for our communication module was designed and field-tested in 2008 and was developed further in the following year.

The selection of course participants for the pilot project in 2008 took place by means of a lottery. Following an introductory event, approximately 150 interested applicants (from 400 students) submitted their names on lists that were made available in the lecture hall, from which 26 participants were selected. Over the course of the supplementary qualification training, one male participant discontinued involvement due to schedule conflicts. In the end, 25 participants participated in the course to its completion, of which 18 (72%) were women and seven (28%) were men. This gender distribution was not representative of the 150 students who volunteered (16% male), but it was nearly representative of the entire cohort (33% male). The average age was 23 years (SD 3 years). In 2009, students were asked to complete a written application, including a short CV and a demonstration of their motivation. From 26 interested students, 20 were selected to participate in the course in 2009, of which 14 (70%) were women and six (30%) were men (full cohort: 39% men). The participants' average age was 24 years (SD 3 years).

All of the students enrolled in the training were in the first year of their degree at the time of participation. The training took place over 19.5 h in 2008 and 33 h in 2009, which progressed over the same time frame as the semester.

To test the effects of the newly developed training against the established, standard course in patient-doctor communication, a comparison group was established. This group included second-year medical students who attended a standard communication course (standard curriculum students = SCS) scheduled in the second year (2008: N = 38, of which 25 (66%) were women and 13 (32%) were men; 2009: N = 13, of which 10 (77%) were women and three (13%) were men).

All students provided informed consent. The study was conducted in full accordance with the 1975 Declaration of Helsinki and the revised version of 1983 and in full accordance with national ethical guidelines.

**Figure 1:**
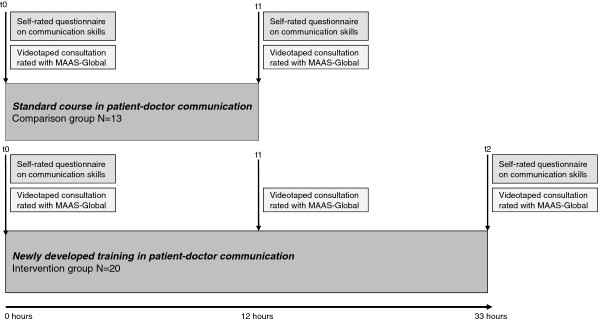
**Summary of study design and measures of the communication module intervention study in 2009**. Note: MAAS-Global = Maastricht History-Taking and Advice Checklist (expert-rated).

Both groups completed self-evaluation questionnaires on their communicative skills at the beginning and end of the course. In 2009, a role-play (approx. five minutes) with simulated patients was videotaped for each student at the beginning (t0) and end (t1 for SCS, t2 for PMS) of the course and was subsequently rated to provide a more objective measure in addition to the self-evaluation questionnaires. The PMS group had another videotaped consultation in the middle of the course (t1). At this measurement point, the number of lessons taught was the same for both groups (see Figure 1).

### Content of the module

Students were given the opportunity to develop their basic communication skills in peer role-playing scenarios and role-playing sessions with simulated patients. The use of simulated patients or actors has proven to be a particularly effective method for training communication skills cf. [30,31]. Parallel to participation in these exercises, the students developed guidelines on physician communication, which covered the core elements in patient-physician communication according to the Kalamazoo Consensus Statement [29]. These guidelines were then used as a basis for further teaching to provide the students with a manageable tool focused on the primary issues of good patient-physician communication. The guidelines included short descriptions of skills relevant to each of the following domains: (1) building the doctor-patient relationship; (2) opening the discussion; (3) handling emotion; (4) exploring details; (5) reaching agreement on further procedures; and (6) summing up the consultation.

During a subsequent lesson, the students led conversations to determine medical histories with simulated patients. An introduction to psycho-oncology provided the students with a glimpse into the emotional reactions of patients with severe physical illnesses through the use of selected case studies, allowing students to broach the issue of dealing with these reactions. Another lesson addressed the conveyance of the pros and cons of illness-related decisions (risk communication). Here too, case studies and video recordings of doctor-patient communication served as the basis for the examination and discussion of the exchange of information concerning prognoses or probabilities of health-related issues. The presentation of case studies through cooperation with a clinic for the treatment of patients with mental illnesses was received particularly well. Three to four students had the opportunity to take a patient's medical history in the presence of a physician. A further conversation with a patient from the oncological outpatient clinic occurred in a subsequent session. These exercises provided the students with the opportunity to utilise and expand upon the conversation techniques acquired during the course. Moreover, the assessment of a psychopathological diagnostic report was practised at the conclusion of the module using the previously discussed case studies.

The content of the module was similar for most of the lessons in 2009, but, in contrast to the limited opportunity in 2008 to conduct a medical history with a patient, all participants had the opportunity to conduct an interview with a patient from a psychosomatic clinic. In preparation for these interviews, students were taught basics of psychosomatic medicine, including common disorders and their prevalence. Furthermore, students practised writing an elaborate case history report for psychosomatic patients.

The course offered to the comparison group was the same in 2008 and 2009. This course comprised four sessions (12 h in total) of communication training, including exercises, peer role play, structured feed-back and encounters with standardised patients (see Table 1).

**Table 1:** Elements of the newly developed training in 2008 and 2009 compared to those of the standard course (number of hours in brackets)

Module "Basics of Communication Skills" (19.5 h) 2008	Module "Basics of Communication Skills" (33 h) 2009	Standard course (12 h) 2008 and 2009
■ Basic introduction: doctor-patient communication (4 h)	■ Basic introduction: doctor-patient communication (4 h)	■ Basic introduction: doctor-patient communication (3 h)

■ Peer role-play (3 h)	■ Peer role-play (2.5 h)	■ Peer role-play (3 h)

■ Simulated patient role-play (3 h)	■ Simulated patients role-play (3 h)	■ Simulated patient role-play (6 h)

■ Introduction to psycho-oncology & interview with a patient (3 h)	■ Writing case history reports in psychosomatics (4.5 h)	

■ Risk communication (3 h)	■ Risk communication (1.5 h)	

Case study presentations in a psychosomatic clinic (3.5 h) ■	Interview with a patient from a psychosomatic clinic ■ (17.5 h)	

### Measurements

Two methods were used to measure improvement in communication skills. First, a self-rated questionnaire was used for evaluation. This questionnaire included two sections: first, a general section on socio-demographic and degree-related information (age, gender, semester and previous professional experience); second, a self-assessment of students' communicative skills and an evaluation of the course lessons. The evaluation was performed on a 6-point Likert scale (1 = very good, 2 = good, 3 = satisfactory, 4 = uncertain, 5 = unsatisfactory to 6 = insufficient). This self-rating was administered to both cohorts: the pilot phase in 2008 and the further evaluation in 2009.

In addition to the questionnaire described above, videotapes were evaluated by trained observers (2 advanced psychology students) using the Maastricht History-Taking and Advice Checklist (MAAS-Global) [32] for the 2009 cohort. The MAAS-Global is a well-established, objective measure for assessing communication skills [33,34], which has been validated in several studies [35,36]. For our study, we chose a modified version of the scale for use with medical students.

The original instrument consisted of 13 items concerning the course of consultation (e.g., *introduction*, *physical examination *or *diagnosis*) and specific communication skills (*exploration, emotion, information giving, summarisations, structuring *and *empathy*). All items concerning the course of consultation were omitted because their content was not relevant to the evaluation of the communication skills course; the primary target of the training was not to conduct an interview in the correct order but to elicit specific communication skills. The items were rated on a 6-point Likert scale (1 = very good, 2 = good, 3 = satisfactory, 4 = uncertain, 5 = unsatisfactory to 6 = insufficient). In addition to these specific items, a subjective overall rating was collected. Raters were blind regarding group membership and the recording time of the videotapes. After completing the individual ratings, consensus ratings for each subject were performed. These ratings were used for all further analyses.

### Statistical analysis

Regarding the reliability of the instrument used, we calculated the intra-class coefficient (ICC) as an adjusted measure of agreement. Fleiss and Cohen [37] showed that the ICC is equivalent to a weighted kappa for measures of reliability, and Landis and Koch [38] provided "rules of thumb" for the interpretation of kappa coefficients. According to these rules, kappa values between .21 and .40 are "fair," those between .41 and .60 are "moderate," those between .61 and .80 are "substantial," and those between .81 and 1.00 are "almost perfect." An examination of the effectiveness of the communication module was performed for both groups (PMS and SCS) using repeated multivariate variance analyses (ANOVA). A partial eta-squared measure was used for the effect size. The interpretation of the effect size is based on the recommendations by Cohen: partial eta-squared < .0099 (small effect), partial eta-squared > .01 and < .0588 (medium effect), and partial eta-squared > .1379 (large effect) [39].

The calculations were performed using SPSS 15 [40].

## Results

### Inter-rater-reliabilities for expert-rated communication skills

Intraclass correlation coefficients (ICCs) between the two advanced psychology students assessing the MAAS-global were in the substantial range for all items except *emotion *(ICC = .85), which showed almost perfect agreement. The level of agreement for the global rating was also found to be almost perfect, showing an ICC of .83.

### Initial group differences

When asked about their motivation to participate in the communication courses, PMS reported significantly higher motivation than SCS in both years of the project (2008: T = -8.1, *p *< .001; 2009: T = -2.4, *p *< .05), and motivation significantly predicted learning success. We found no significant differences between PMS and SCS in expert-rated communication skills at the beginning of the courses (see Table 2). The overall communication skills of both groups (SCS and PMS) were in the moderate-to-fair range prior to the lessons. The most critical issue was *dealing with emotion*. According to this item, skills appeared to be poor in both groups (5.25 for PMS and 4.58 for SCS).

**Table 2:** Means and standard deviations of expert-rated communication skills prior to the lessons in the 2009 cohort (rating scale: 1 = very good; 6 = insufficient)

Item	group	M	SD	t value	p
exploration	PMS^†^	3.50	.95	.00	1.00
		
	SCS	3.50	1.09		

emotion	PMS	5.25	1.21	1.43	.16
		
	SCS	4.58	1.38		

summarisations	PMS	3.60	1.31	-0.27	.79
		
	SCS	3.75	1.86		

structuring	PMS	2.80	.83	.18	.86
		
	SCS	2.75	.62		

empathy	PMS	3.30	.98	.88	.39
		
	SCS	3.00	.85		

**global**	**PMS**	**3.60**	**.82**	**.31**	**.76**
		
	**SCS**	**3.50**	**1.00**		

^† ^*PMS *psychosocial medicine students, *SCS *standard curriculum students

### Effectiveness of the communication training

#### Expert rating of communication skills

**Figure 2:**
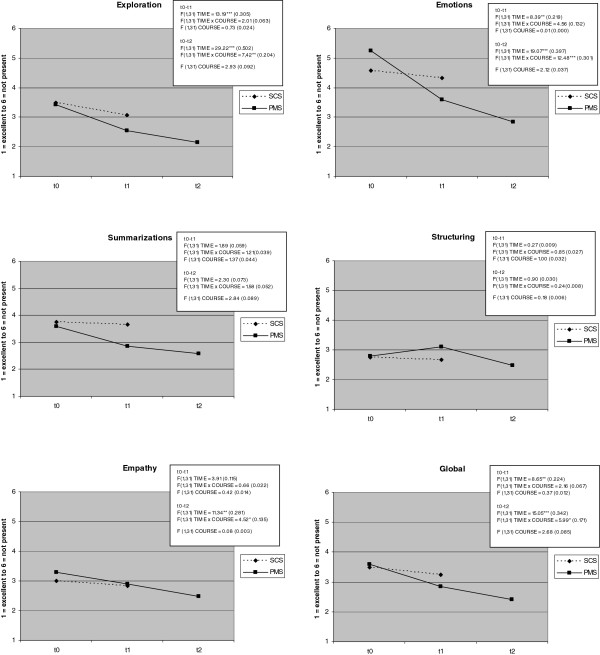
**MAAS-Global rating of communicative competence before and after participation in the seminar: psychosocial medicine students (PMS) vs. standard curriculum students (SCS) at equal duration (t0-t1) and longer duration for PMS (t0-t2), F values and effect sizes (partial Eta-squared) are displayed in the boxes**.

Results from the MAAS-Global video ratings comparing student communication skills at the beginning (t0), middle (t1 for PMS) and end (t1 for SCS) of the course are displayed in Figure 2. Although a significant improvement of the global appraisal and the items *exploration *and *emotion *were observed over the duration of the course for both groups, a significant interaction effect was only found for *emotion *(F_(1,31) TIME × COURSE _= 4.56; *p *< .01; partial ε^2 ^= .132). There is not only a significant increase over time, but PMS students also improve more than the controls within the same time interval. Nevertheless, all remaining effect sizes for interaction effects, including global appraisal, are in the medium range (partial eta-squared > .01 and < .0588, see Figure 2).

Considering the third point of measurement (end of PMS course) in the comparison of SCS and PMS communication skills, global improvement due to the duration of the course becomes more apparent (F_(1,30) TIME _= 15.05, *p *< .001, ε^2 ^= .342), and the interaction effect gains significance (F_(1,30) TIME × COURSE _= 5.99; *p *< .05; ε^2 ^= .171). The number of items showing interaction effects also increases (see Figure 2). Improvements in *explorative behaviour *and *empathy *are more apparent for PMS than SCS if the entire duration of the newly developed training is considered.

#### Self-rating of communication skills

In the pilot phase, the communication skills of the SCS (n_min _= 38) barely improved, whereas the self-assessments completed by the PMS (n_min _= 22) exhibited significant improvement after participating in the course. For the course in 2009, similar effects were identified. Again, the PMS (n_min _= 20) had the impression, that they profited more from the newly developed communication training than the SCS (n_min _= 12) did from the standard training (see Table 3).

**Table 3:** Self-assessment of communicative competence before and after participation in the seminar (rating scale: 1 = very good; 6 = insufficient)

	Year	Group	Pre (t0) M (SD)	Post (t1) M (SD)	F value (Partial eta-squared)
communicative competence	2008	PMS^†^	2.13 (.63)	1.61 (.50)	F_(1,72) TIME _= 4.08* (.054)
		
		SCS	2.02 (.68)	2.12 (.93)	F_(1,72) TIME × COURSE _= 8.73** (.108)

					F _(1,72) COURSE _= 1.64 (.022)

communicative competence	2009	PMS	2.45 (.51)	1.90 (.55)	F_(1,30) TIME _= .41 (.014)
		
		SCS	2.58 (.90)	2.92 (1.08)	F_(1,30) TIME × COURSE _= 6.85* (.187)

					F _(1,30) COURSE _= 7.52* (.201)

†*PMS *psychosocial medicine students; *SCS *standard surriculum students; *significant at the 5% level, **significant at the 1% level

#### Relationship between self- and expert-rated measurements of students' communication skills

In general, no significant correlation between self-administered communicative competence and expert-rated overall communication skills was identified. Students from both groups tended to overestimate their competencies at the beginning of the lessons. Within the PMS group, there was a tendency toward increasing agreement between self- and expert-rated skills and between the beginning (r = .08) and end of the course (r = .36). For the SCS group, an opposite effect was identified (t0: r = .28; t1: r = .07).

### Overall evaluation of the supplementary qualification training

The PMS rated the module "Basics and Practice in Communication Skills" as very satisfactory in the 2008 pilot phase. The module was rated very well by the students with a mean value of 1.6 (SD = .2) (scale: 1 = very good; 6 = unsatisfactory). In the evaluation of the module at the conclusion of the course, students indicated that they would like to see more detailed and advanced exercises in future courses. They also expressed an interest in further opportunities to practice the role of the physician in role-playing scenarios. These suggestions were considered during the conceptual development of the course, which resulted in a slightly better student rating in 2009 (M = 1.2; SD = .4). In both years, patient interviews were rated best, compared to other contents of the courses (2008: M = 1.3; SD = 0.9, 2009: M = 1.1; SD = 0.2).

## Discussion

The objective of the present study was the development, implementation, and evaluation of a course in communication skills for medical students and the identification of the most effective components for future courses in the new curriculum at our medical school.

With respect to content, we referred to the Kalamazoo Consensus Statement [29]. Based on an extensive review of the literature and our experiences with standard communication courses offered at the University Medical Centre Hamburg-Eppendorf, we considered the aspects described within this statement an appropriate and useful framework for developing a new training program in communication skills.

The initial evaluation of the program was conducted during the pilot phase of the study in 2008 and was continued the following year. The results from the first year of the program demonstrate that the psychosocial medicine students (PMS) showed a considerable increase in subjective perceived skills and expertise after participating in the training. It was also observed that the PMS surpassed the medical students in the comparison group with respect to the self-assessment of their communication skills. These findings were also evident in the following year and were validated by an additional standardised peer assessment of communication skills.

Regarding the results from the expert-rated measure, the PMS showed significant improvements compared to the SCS after participation in the communication module with regard to the global appraisal and three of five aspects (*exploration*, *emotion *and *empathy*). It should be noted that the significance of the interaction effects depends on the point of measurement considered. Comparing PMS and SCS at similar points in the course, only one interaction effect was significant (*emotion*), whereas at a longer duration for PMS, two additional effects as well as the global appraisal item gain significance. At first sight, this suggests that the number of lessons is a crucial factor in the effectiveness of the training. On closer inspection of the supplementary course between the second and the last point of measurement, it strikes out that 70% of the additional lessons were covered in patient interviews in a real-world setting, leading to the assumption that not only time but also practice is important;. These findings are in line with the results of Hulsman et al. [41], who found training embedded in clinical contexts to be more effective than training with a more theoretical focus. It can further be assumed that the remaining two aspects (*structuring, summarisations*) were not the main focus of the training and involved more technical communicative skills that should be given detailed attention in an advanced training course.

The study's relatively small sample size allowed for only a limited interpretation of the results. Another limitation of our study design was the constitution of comparison groups. For practical reasons, we were not able to randomly assign students from the same year to an intervention and a control group, and we had to ask students from standard communication courses in the second year of their studies to participate as controls. There is a difference in general experiences during their prior medical education between the two groups, which might have an impact on the results of our study. Therefore, we were only able to provide preliminary data on the superiority of the newly developed training due to the possibility of group bias. The participants in the intervention group volunteering to take part in the project might also have an impact on the results. For example, students from the intervention group had greater motivation to attend the course than students from the standard curriculum, which influenced learning success. This result must be taken into account when interpreting the efficacy of the training. Future examinations of training effects should include a randomised controlled design. It is also advisable to review the effects of the psycho-med supplementary qualification training over a longer time period, which would facilitate assertions about any lasting effects of this study. One possible example of such an assessment would be an accompanying evaluation of the PMS until their completion of the degree program. Moreover, as the inter-rater reliabilities of the MAAS-Global items are considered to be nearly perfect for only one of five items, an expanded training and supervision of the ratings should be applied in further evaluation studies.

With regard to the agreement between self- and expert-rated communication skills, we found no significant correlation in our study, which corresponds to the results reported by Millis et al. [42]. These authors identified a low level of agreement between simulated patient ratings and resident physicians' self-ratings of interpersonal skills based on a short history-taking interview. In contrast, a recent study by Cave et al. [43] found a significant correlation between self-assessment, peer scores and tutor scores in a sample of third-year medical students. The authors assumed that the students' ability to assess their own performance in communication skills might be influenced by the students being trained to use the assessment criteria and given anchor statements. A similar effect might explain the observed trend of an increasing positive relation between the MAAS-Global rating and the self-assessment of communication skills in the PMS group. In the course of the training, students became familiar with aspects of good patient-physician communication. Continuous feedback from tutors, peers and trainers might have contributed to more elaborate self-reflection and resulted in more realistic self-evaluations of students' competencies. Comparable experiences within the standard course might have been less distinct, resulting in a moderate decrease in the observed correlation between self- and expert-rated skills.

## Conclusions

Overall, the results of the present study indicate the usefulness of communication training within the undergraduate curriculum. From this study, we have learned that the potential for students to exercise newly acquired skills in a real-world setting is the most favoured and probably the most effective method of teaching communication, and elaborate self-reflection consolidates learning success. We think it is worthwhile to offer students the possibility to reflect on and practise their communication skills at an early stage of their degree program as this approach will enable them to acquire and expand these competencies throughout the curriculum for later use. These findings and experiences will be used to develop and implement communication training in the reformed curriculum at the University Centre Hamburg-Eppendorf.

## Competing interests

The authors declare that they have no competing interests.

## Authors' contributions

All authors contributed to the design of the study, the collection and interpretation of data and the drafting of the article. MCH performed the statistical analysis. All authors read and approved the final manuscript.

## Pre-publication history

The pre-publication history for this paper can be accessed here:

http://www.biomedcentral.com/1472-6920/12/16/prepub

## References

[B1] Maguire P Pitceathly C Key communication skills and how to acquire them BMJ 2002 325 697 700 10.1136/bmj.325.7366.697 12351365 PMC1124224

[B2] Clever SL Jin L Levinson W Meltzer DO Does doctor-patient communication affect patient satisfaction with hospital care? Results of an analysis with a novel instrumental variable Health Serv Res 2008 43 1505 1519 10.1111/j.1475-6773.2008.00849.x PMC2653895 18459954

[B3] Parle M Jones B Maguire P Maladaptive coping and affective disorders in cancer patients Psychol Med 1996 26 735 744 10.1017/S0033291700037752 8817708

[B4] Ramirez AJ Graham J Richards MA Cull A Gregory WM Mental health of hospital consultants: the effects of stress and satisfaction of work Lancet 1995 347 724 728 8602002 10.1016/s0140-6736(96)90077-x

[B5] Roter DL Hall JA Kern DE Barker LR Cole KA Roca RP Improving physicians' interviewing skills and reducing patients' emotional distress Arch Intern Med 1995 155 1877 1884 10.1001/archinte.1995.00430170071009 7677554

[B6] Bertakis KD Azari R Patient-centered care is associated with decreased health care utilization J Am Board Fam Med 2011 24 229 239 10.3122/jabfm.2011.03.100170 21551394

[B7] Whitehouse CR The teaching of communication skills in United Kingdom medical schools Med Educ 1991 25 311 318 10.1111/j.1365-2923.1991.tb00072.x 1890961

[B8] Makoul G The SEGUE Framework for teaching and assessing communication skills Patient Educ Couns 2001 45 23 34 10.1016/S0738-3991(01)00136-7 11602365

[B9] Rider EA Hinrichs MM Lown BA A model for communication skills assessment across the undergraduate curriculum Med Teach 2006 28 127 134 10.1080/01421590600726540 16973446

[B10] Brown J How clinical communication has become a core part of medical education in the UK Med Educ 2008 42 271 278 10.1111/j.1365-2923.2007.02955.x 18275414

[B11] von Fragstein M Silverman J Cushing A Quilligan S Salisbury H Wiskin C UK consensus statement on the content of communication curricula in undergraduate medical education Med Educ 2008 42 1100 11017 10.1111/j.1365-2923.2008.03137.x 18761615

[B12] Deveugele M Derese A De Maess-chalck S Willems S Van Driel M De Maeseneer J Teaching communication skills to medical students, a challenge in the curriculum? Patient Educ Couns 2005 58 265 270 10.1016/j.pec.2005.06.004 16023822

[B13] van Dalen J Kerkhofs E van Knippenberg-Van Den Berg BW van Den Hout HA Scherpbier AJ van der Vleuten CP Longitudinal and concentrated communication skills programmes: two dutch medical schools compared Adv Health Sci Educ 2002 7 29 40 10.1023/A:1014576900127 11912332

[B14] Bosse HM Nickel M Huwendiek S Jünger J Schultz JH Nikendei C Peer role-play and standardised patients in communication training: a comparative study on the student perspective on acceptability, realism, and perceived effect BMC Med Educ 2010 10 27 10.1186/1472-6920-10-27 20353612 PMC2853557

[B15] Jünger J Köllner V Integration von Kommunikationstrainings in die klinische Lehre [Intergration of a doctor-patient communication training into clinical teaching] Psychother Psych Med 2003 53 47 55 10.1055/s-2003-36965 12552412

[B16] Jünger J Schäfer S Roth C Schellberg D Friedman Ben-David M Christoph N Effects of basic clinical skills training on objective structured clinical examination performance Med Educ 2005 39 1015 1020 10.1111/j.1365-2929.2005.02266.x 16178828

[B17] Langewitz WA Eich P Kiss A Wossmer B Improving communication skills--a randomized controlled behaviorally oriented intervention study for residents in internal medicine Psychosom Med 1998 60 268 276 9625213 10.1097/00006842-199805000-00009

[B18] Jungbauer J Alfermann D Kamenik C Brähler E Vermittlung psychosozialer Kompetenzen mangelhaft [Psychosocial skills training unsatisfactory] Psychother Psych Med 2003 2003 319 321 10.1055/s-2003-40493 12847666

[B19] Kiessling C Dieterich A Fabry G Hölzer H Langewitz W Mühlinghaus I Pruskil S Scheffer S Schubert S Communication and social competencies in medical education in German-speaking countries: the basel consensus statement. Results of a Delphi survey Patient Educ Couns 2010 81 259 266 10.1016/j.pec.2010.01.017 20223614

[B20] Haidet P Dains JE Paterniti DA Hechtel L Chang T Tseng E Rogers JC Medical student attitudes toward the doctor-patient relationship Med Educ 2002 36 568 574 10.1046/j.1365-2923.2002.01233.x 12047673

[B21] Silverman J Teaching clinical communication: A mainstream activity or just a minority sport? Patient Educ Couns 2009 76 361 367 10.1016/j.pec.2009.06.011 19647971

[B22] Losh DP Mauksch LB Arnold RW Maresca TM Storck MG Maestas RR Goldstein E Teaching inpatient communication skills to medical students: an innovative strategy Acad Med 2005 80 118 124 10.1097/00001888-200502000-00002 15671313

[B23] Wahlqvist M Mattsson B Dahlgren G Hartwig-Ericsson M Henriques B Hamark B Hösterey-Ugander U Instrumental strategy: a stage in students' consultation skills training? Scand J Prim Health Care 2005 23 164 170 10.1080/02813430510018646 16162469

[B24] Zick A Granieri M Makoul G First-year medical students' assessment of their own communication skills: a video-based, open-ended approach Patient Educ Couns 2008 68 161 166 17640843 10.1016/j.pec.2007.05.018

[B25] Harrison A Glasgow N Townsend T Communication skills training early in the medical curriculum: the UAE experience Med Teach 1996 18 35 41 10.3109/01421599609040260

[B26] Humphris GM Kaney S Assessing the development of communication skills in undergraduate medical students. [see comment] Med Educ 2001 35 225 231 10.1046/j.1365-2923.2001.00869.x 11260445

[B27] Shapiro SM Lancee WJ Richards-Bentley CM Evaluation of a communication skills program for first-year medical students at the University of Toronto BMC Med Educ 2009 9 11 10.1186/1472-6920-9-11 19232138 PMC2654445

[B28] Cegala DJ Lenzmeier Broz S Physician communication skills training: a review of theoretical backgrounds, objectives and skills Med Educ 2002 36 1004 1016 10.1046/j.1365-2923.2002.01331.x 12406260

[B29] Makoul G Essential elements of communication in medical encounters: the Kalamazoo consensus statement Acad Med 2001 2001 390 393 11299158 10.1097/00001888-200104000-00021

[B30] Evans R Elwyn G Edwards A Review of instruments for peer assessments of physicians Br Med J 2004 328 1240 1244 10.1136/bmj.328.7450.1240 15155502 PMC416602

[B31] Wind LA von Dalen J Muijtjens AMM Rethans JJ Assessing simulated patients in an educational setting:the MaSP (Maastricht Assessment of Simulated Patients) Med Educ 2004 38 39 44 10.1111/j.1365-2923.2004.01686.x 14962025

[B32] van Thiel J Ram P van Dalen J MAAS-Global Manual 2000 Maastricht University

[B33] Boon H Stewart M Patient-physician communication assessment instruments: 1986 to 1996 in review Patient Educ Couns 1998 35 161 176 10.1016/S0738-3991(98)00063-9 9887849

[B34] Schirmer JM Mauksch L Lang F Marvel MK Zoppi K Epstein RM Brock D Pryzbylski M Assessing communication competence: a review of current tools Fam Med 2005 37 184 192 15739134

[B35] Ram P Grol R Rethans JJ Schouten B van der Vleuten M Kester A Assessment of general practitioners by video observation of communicative and medical performance in daily practice: issues of validity, reliability and feasibility Med Educ 1999 33 447 454 10.1046/j.1365-2923.1999.00348.x 10354322

[B36] van Thiel J Kraan CP van der Vleuten M Reliability and feasibility of measuring medical interviewing skills: the revised Maastricht history-taking and advice checklist Med Educ 1991 25 224 229 10.1111/j.1365-2923.1991.tb00055.x 1857278

[B37] Fleiss JL Cohen J The equivalence of weighted kappa and the intraclass correlation coefficient as measures of reliability Educ Psychol Meas 1973 33 613 619 10.1177/001316447303300309

[B38] Landis JR Koch GG The measurement of observer agreement for categorical data Biometrics 1977 33 159 174 10.2307/2529310 843571

[B39] Cohen J Statistical power and analysis for the behavioral sciences 1988 2 Hillsdale: Lawrence

[B40] SPSS SPSS Version 15.0 for Windows 2009 Chicago: SPSS

[B41] Hulsman RL Ros WJG Winnubst JAM Bensing JM Teaching clinically experienced physicians communication skills. A review of evaluation studies Med Educ 1999 33 655 668 10.1046/j.1365-2923.1999.00519.x 10476016

[B42] Millis SR Jain SS Eyles M Tulsky D Nadler SF Foye PM Elovic E DeLisa JA Assessing physicians' interpersonal skills do patients and physicians see eye-to-eye? Am J Phys Med Rehabil 2002 81 946 951 10.1097/00002060-200212000-00011 12447094

[B43] Cave J Washer P Sampson P Griffin M Noble L Explicitly linking teaching and assessment of communication skills Med Teach 2007 29 317 322 10.1080/01421590701509654 17786744

